# Boron accumulation by *Lemna minor* L. under salt stress

**DOI:** 10.1038/s41598-018-27343-y

**Published:** 2018-06-12

**Authors:** Chunguang Liu, Wancong Gu, Zheng Dai, Jia Li, Hongru Jiang, Qian Zhang

**Affiliations:** 1 Key Laboratory of Pollution Processes and Environmental Criteria (Ministry of Education), Nankai University, Tianjin, 300350 China; 2 Tianjin Key Laboratory of Environmental Remediation and Pollution Control, Tianjin, 300350 China

## Abstract

Excess boron (B) is toxic to aquatic organisms and humans. Boron is often present in water with high salinity. To evaluate the potential of duckweed (*Lemna minor* L.) for removing B from water under salt stress, we cultured duckweed in water with 2 mg/L of B and sodium chloride (NaCl) concentrations ranging from 0 to 200 mM for 4 days. The results show that with increasing salinity, the capacity of *L. minor* to accumulate B initially decreased and then increased. *L. minor* used different mechanisms to accumulate boron at lower and higher levels of salt stress. The growth and chlorophyll synthesis of *L. minor* were significantly inhibited when the concentration of NaCl reached 100 mM. Our results suggest that *L. minor* is suitable for the accumulation of B when NaCl salinity is below 100 mM.

## Introduction

Boron (B), an essential element for plant growth, is often present in excessive concentrations in industrial wastewater, mine effluent, and irrigation water^1–3^. High concentrations of B in water may exert a negative impact on aquatic organisms and can pose a potential health hazard to humans and animals^4,5^. B removal efficiency is poor in conventional water treatment systems^6^, leading to the development of various specific B removal techniques, including precipitation-coagulation, ion exchange, solvent extraction, ultrafiltration, and adsorption with B-selective resins^7^. Unfortunately, most of these methods are associated with high operation and maintenance costs, as well as the overuse of chemicals^8^. Thus, it is necessary to explore simple, inexpensive, and environmentally friendly technologies for removing B from water.

Duckweed is a small, free-floating aquatic angiosperm that grows throughout much of the world^9^, and it has been considered as a potential candidate for B removal^3,4,10^. Previous studies have shown the ability of duckweed to tolerate and remove B under different conditions. For example, *Lemna minor*, a widespread species of duckweed, was shown to tolerate and accumulate B differently under various conditions^11^. *Spirodela polyrrhiza*, another species of duckweed (also called greater duckweed), reportedly had different growth responses to B toxicity at different initial B concentrations^12^. *Lemna gibba*, also a widespread variety of duckweed, was observed to efficiently remove B at concentrations below 2 mg/L^8^. In another study, however, *L. gibba* was found to be suitable for remediating B-contaminated water at B concentrations of 10 and 25 mg/L^1^.

Boron is often found at high concentrations in association with other salts in saline irrigation water^13,14^. Excess salt in water can decrease the osmotic potential of water, resulting in oxidative stress on plants^15^. Sodium chloride (NaCl) is recognized as the most common salt^16^. Sodium is not essential for plants but is toxic and often induces cellular damage that inhibits plant growth and development^17^. Growth inhibition from salt stress has been reported for several species of duckweeds including *S. polyrhiza*, *L. minor*, and *L. gibba*^18^. The removal of pollutants (e.g., technetium, nickel, and cadmium) by duckweed was observed to decrease under salt stress^19–21^. However, the influence of salt stress on the ability of duckweed to remove B is still unknown.

The purpose of the present work is to evaluate the performance of the duckweed species *L. minor* in B removal under salt stress. To this end, we cultivated *L. minor* in water with 2 mg/L of B and NaCl ranging from 0 to 200 mM. We tested the changes in B concentration in the water, the accumulation of B, Na, and K in plant tissue, and the growth and chlorophyll synthesis of *L. minor*. On the basis of this research, we evaluated the potential of duckweed as a candidate for removing B from water under salt stress.

## Materials and Methods

### Plant cultivation

*L. minor* colonies were isolated from a lake in Xiqing District of Tianjin, China and were cultured aseptically in half-Hoagland’s solution. The plant was acclimated for one week prior to the formal experiment.

### Batch experiment

The acclimated *L. minor* colonies were transferred to a polypropylene container (15 × 10.5 × 7.5 cm^3^) filled with 750 ml of half-Hoagland’s solution. Two grams (fresh weight) of duckweed was cultivated in each container. The boron concentration was set at 2 mg/L by adding boric acid (H_3_BO_3_) with a concentration of 0.25 mg/L as B into half-Hoagland’s solution. NaCl was added to the B-laden solution to generate five concentrations: 0, 50, 100, 150, and 200 mM. One container was filled with 750 ml of half-Hoagland’s solution (with 2 mg/L of B and without NaCl) and left unplanted to serve as a control. Each treatment was replicated four times. The plant was cultivated in a culture room with a photoperiod of 16:8 and light intensity of 72 µmol/m^2^/s. During the experiment, the air temperature ranged from 19 to 26 °C. The experiment lasted for four days. Water loss by evapotranspiration was monitored and corrected by weighing the containers each day and adding deionized water.

### Water and plant sampling and analysis

Five millilitres of water was collected from each container at time periods of 1, 2, and 4 days for measurement of B. The water samples for B determination were filtered through a 0.45-µm membrane and then stored in a centrifuge tube at 4 °C.

At the end of the cultivation experiment, the plants were harvested and rinsed with deionized water. Residual water on the duckweed was removed using tissue paper. Each container of duckweed was weighed to determine the fresh weight (FW), and 0.1 g of the fresh sample was subsequently transferred to a centrifuge tube for chlorophyll determination. The remainder of each sample was dried at 80 °C for 48 h to obtain the dry weight (DW).

For chlorophyll determination, 0.1 g of fresh duckweed sample was extracted with 10 ml of 95% ethanol (v/v) for 72 h at room temperature in the dark. The extract was then centrifuged at 2790 × *g* for 10 min, and the absorbance of the supernatant was determined at 663 and 645 nm using a spectrophotometer (T6, Persee General, Beijing, China), using the method described by Huang *et al*.^22^. Chlorophyll was calculated using the following equations:

1 $${C}_{a}=12.72{A}_{663}-2.69{A}_{645}$$

2 $${C}_{b}=22.90{A}_{645}-4.68{A}_{663}$$

3 $${C}_{chl}={C}_{a}+{C}_{b}$$

where *C*_*a*_, *C*_*b*_, and *C*_*chl*_ represent the contents of chlorophyll *a*, chlorophyll *b*, and total chlorophyll, respectively; *A*_663_ and *A*_645_ are the absorbances at 663 and 645 nm, respectively.

Each dried sample was separately ground into powder using a mortar and pestle. The ground duckweed samples were digested using a graphite digester (SH220N, Hanon Instruments, Jinan, China) according to Kaur *et al*.^23^ and Liu *et al*.^24^ with minor modifications. Aliquots (0.100 g) of ground samples were digested with 5 ml of nitric acid (HNO_3_) and 1 ml of 30% hydrogen peroxide (H_2_O_2_) at 90 °C for 4 h. The digested solution was filtered through a 0.45-µm membrane and then diluted to 25 ml with deionized water. Boron concentrations of the digested solutions and the water samples were determined using inductively coupled plasma-optical emission spectrometry (ICP-OES) (PS-I, Teledyne Leeman Labs, Hudson, NH, USA).

The B mass balance analysis was performed for the treatment system as follows:

4 $${B}_{t}={B}_{s}+{B}_{p}+{B}_{o}$$

where *B*_*t*_ is the total B (mg) in the cultivation system; *B*_*s*_ is the water-soluble B (mg); *B*_*p*_ is the plant-accumulated B (mg); and *B*_*o*_ is other forms of B (mg).

5 $${B}_{t}={C}_{i}\times {Q}_{i}$$

where *C*_*i*_ is the initial B concentration (mg/L) of the water and *Q*_*i*_ is the initial water volume (L).

6 $${B}_{p}={C}_{p}\times {W}_{d}$$

where *C*_*p*_ is the B concentration (mg/g) of the plant tissue and *W*_*d*_ is the dry weight (g) of duckweed.

7 $${B}_{s}={C}_{{\rm{w}}}\times {Q}_{f}$$

where *C*_*w*_ is the final B concentration (mg/L) of the water and *Q*_*f*_ is the final water volume (L). Thus,

8 $${B}_{o}={B}_{t}-{B}_{s}-{B}_{p}$$

### Statistics

Four independent replications were used for each treatment, and the error bars are presented as the mean ± standard deviation. Data were analysed using a one-way ANOVA followed by Duncan’s multiple range test (*p* < 0.05).

## Results and Discussion

### Growth of duckweed

**Figure 1:**
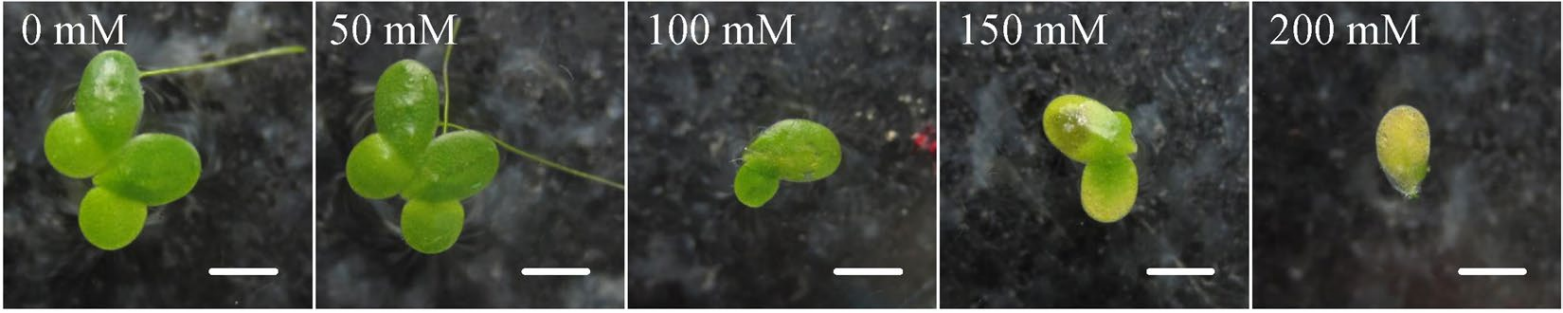
Morphological characteristics of *L. minor* at different concentrations of NaCl (Bar = 2 mm).

The growth of *L. minor* was inhibited by NaCl, and visible damage appeared in the fronds at high NaCl concentrations. At the end of the experiment, a normal green colour was observed on the fronds of *L. minor* grown at NaCl concentrations between 0 and 50 mM (Fig. 1). Four fronds were observed on most duckweed plants grown at NaCl concentrations between 0 and 50 mM. In the 100 mM NaCl treatment, only two fronds with slight chlorosis were observed on most plants. In the 150 mM NaCl experiment, two fronds and a green-yellow colour were recorded for most plants. In the 200 mM NaCl treatment, most plants had only one frond and were severely chlorotic and even bleached. In the 100, 150, and 200 mM NaCl treatment, the duckweed roots became very fragile and easily dropped from the fronds. These results are consistent with the previous studies on duckweed under salt stress^16,25^. The chlorosis is mainly attributed to the salt-induced oxidative damage, which degrades chloroplasts in the duckweed fronds^26^. The plasma membrane of duckweed can be damaged by severe salt stress, resulting in fragile stipes and fallen fronds^27^.

**Figure 2:**
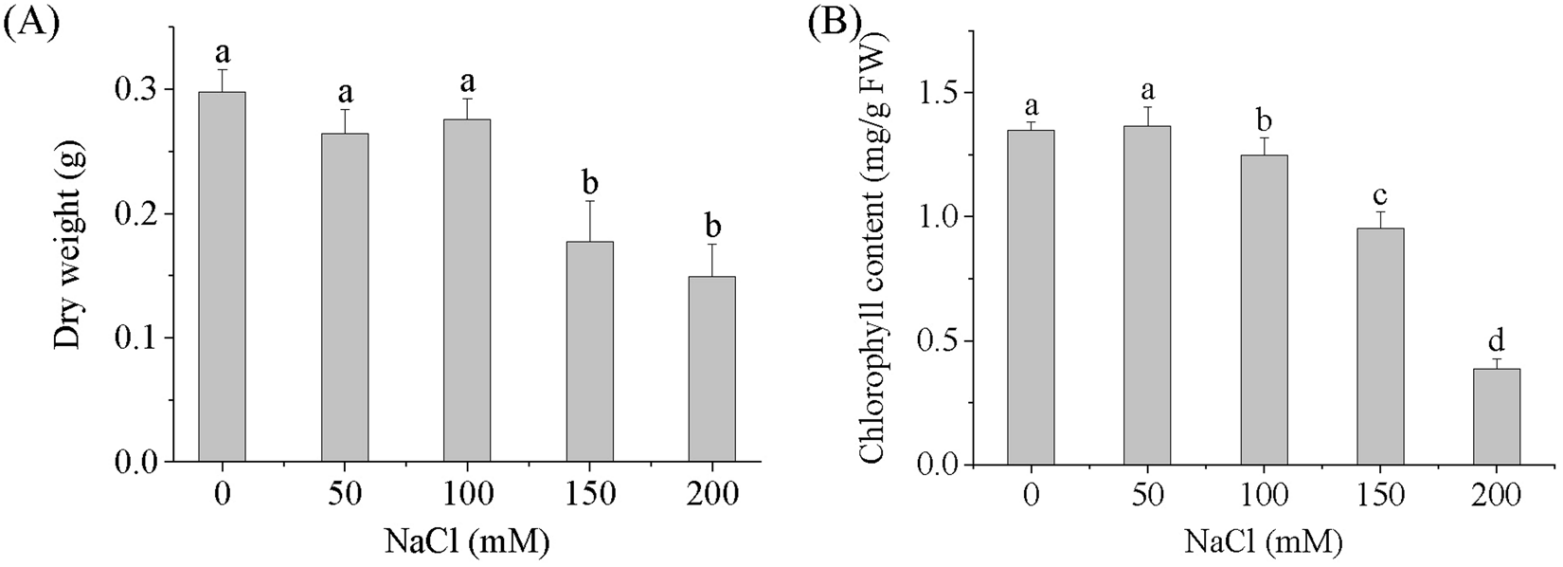
Effect of NaCl on dry weight (**A**) and chlorophyll content (**B**) of *L. minor*. Values shown are the average ± standard deviation of four replicates. Values with different letters are significantly different (*p* < 0.05).

The dry weight of duckweed plants decreased gradually with increasing NaCl concentrations (Fig. 2(A)). Compared with the control, however, no significant decrease in the dry weight of duckweed was observed in the plants grown in 50 and 100 mM NaCl solutions. Significant decreases in dry weight were found in the plants grown in 150 and 200 mM NaCl. These results indicate that in terms of biomass accumulation, duckweed is able to tolerate 100 mM NaCl. A previous study reported that *L. minor* grew well in a 62.5 mM NaCl solution, but growth was inhibited in solutions with NaCl concentrations of 125 mM and higher^28^. We recently observed that the biomass of *L. minor* decreased significantly in solutions with NaCl concentrations of 50 mM and higher^27^. The higher tolerance of *L. minor* to NaCl can possibly be attributed to the presence of B, which is able to inhibit the uptake and accumulation of Cl^−^, a major toxic ion in plants^14^.

To further understand the effect of NaCl stress on the growth of *L. minor*, the content of chlorophyll was determined at the end of the cultivation. As shown in Fig. 2(B), there was no significant difference in the chlorophyll content of the control and the plant grown in a 50 mM NaCl solution. Compared to the control, chlorophyll contents of the plants grown in the 100, 150, and 200 mM NaCl solutions decreased significantly. Excess NaCl can damage the chloroplast envelope and thylakoid through increased production of free radicals, resulting in the decrease of chlorophyll *a* and *b* production^29^. Chlorophyll in *L. minor* reportedly decreases gradually with increasing NaCl and significantly decreases when NaCl reaches 8 g/L (136.9 mM)^25^. In the present work, plants grown in solutions with NaCl concentrations of 100 mM and higher also showed a significant decrease in chlorophyll content, suggesting that the chloroplasts of *L. minor* were damaged by solutions with 100 mM NaCl concentrations.

### Boron accumulation in duckweed

Boron concentrations in duckweed tissue were determined after 4 days of cultivation (Table 1). Boron concentrations in duckweed tissue progressively decreased with increasing NaCl concentrations in the 0 to 100 mM concentration range. However, when NaCl concentrations were increased to 150 and 200 mM, B concentrations in *L. minor* increased to the levels measured in the control. B uptake is a passive process via mass flow within the transpiration stream for most higher plants with adequate and excessive B supply^30,31^. Excess salt lowers the osmotic potential of water and inhibits transpiration, which reduces the absorption of B by the plant^14^. This mechanism explains the decrease in B uptake by *L. minor* in solutions with NaCl concentrations of 50 and 100 mM. At higher NaCl concentrations, the increase in B accumulation is attributed to damage of the cell membrane of *L. minor* induced by salt stress. The integrity of plant cell membranes is important for the plant to restrict the uptake of excess B^32^. Salt-induced loss of membrane integrity and an increase in membrane permeability were also found for duckweed examined in previous studies^27,33^. An increase in cell membrane permeability allows more B to enter the duckweed cell via passive diffusion^34^.

**Table 1:** Effect of NaCl on bioconcentration factor (BCF) of B in *L. minor*.

NaCl (mM)	B in duckweed (mg/g)	BCF
0	0.78 ± 0.11 ab	391.03 ± 55.31 ab
50	0.55 ± 0.05 c	273.94 ± 25.31 c
100	0.43 ± 0.08 d	213.44 ± 37.91 d
150	0.73 ± 0.04 b	367.22 ± 19.04 b
200	0.85 ± 0.06 a	424.13 ± 30.19 a

Values shown are the average ± standard deviation of four replicates. Values with different letters are significantly different (*p* < 0.05).

The bioconcentration factor (BCF) of B in *L. minor* was calculated and is shown in Table 1. The trend of BCF values with increasing NaCl concentrations were similar to B concentrations in duckweed tissue. At NaCl concentrations of 0 and 200 mM, there was no significant difference in the BCF values of *L. minor*, which reached 391.03 and 424.13, respectively. These values are close to those in a previous study^4^, in which the BCF of B obtained for *L. minor* and *L. gibba* was ~350 and ~425 respectively, at 2 mg B/L. Two criteria, the BCF and the translocation factor (TF, shoot or leaf concentration/root concentration), have been used to identify the hyperaccumulation of an element^35,36^. When both BCF and TF exceed 1.0, hyperaccumulation is thought to occur^37^. Since duckweed absorbs nutrients through all surfaces including fronds and roots, TF is not suitable for evaluating the accumulation capacity of duckweed. In the present study, the BCF values of B in duckweed are much greater than 1.0, indicating a strong capacity for B accumulation.

### Boron mass balance and distribution in the system

In duckweed treatment systems, pollutants may be removed by absorption, adsorption, or precipitation, and mass balance analyses are widely used to investigate the fate of the pollutants^38^. The mass balance of B was calculated and is shown in Table 2. It can be seen that only 7.9% to 15.5% of B was accumulated by *L. minor*, indicating that a limited proportion of B was removed by harvesting. The maximum accumulation (15.5%) was observed at a NaCl concentration of 0 mM, followed by a NaCl concentration of 50 mM (9.7%). Under NaCl concentrations ranging from 100 to 200 mM, B accumulation was much lower. In 0 to 100 mM NaCl solutions, water-soluble B was not significantly modified by increased NaCl. In 150 and 200 mM NaCl solutions, however, water-soluble B decreased dramatically. The insoluble forms of B increased gradually with the increased NaCl concentration. It should be noted that the percentage of insoluble B even reached 63.1% in the 200 mM NaCl solution, suggesting that a major proportion of B was transformed into insoluble forms.

**Table 2:** Mass balance of B in *L. minor* treatment system.

NaCl (mM)	Total B (mg)	Accumulated B (mg)	Soluble B (mg)	Insoluble B (mg)
0	1.5 (100%)	0.233 ± 0.038 a (15.5%)	1.024 ± 0.039 ab (68.3%)	0.243 ± 0.033 c (16.2%)
50	1.5 (100%)	0.145 ± 0.021 b (9.7%)	1.082 ± 0.088 a (72.1%)	0.273 ± 0.069 c (18.2%)
100	1.5 (100%)	0.118 ± 0.022 b (7.9%)	1.085 ± 0.096 a (72.4%)	0.297 ± 0.106 c (19.8%)
150	1.5 (100%)	0.129 ± 0.019 b (8.6%)	0.887 ± 0.132 b (59.2%)	0.483 ± 0.150 b (32.2%)
200	1.5 (100%)	0.126 ± 0.022 b (8.4%)	0.427 ± 0.156 c (28.4%)	0.947 ± 0.148 a (63.1%)

The values except total B represent the mean of four replicates ± standard deviation. Means followed by the same letter in the same column do not differ significantly according to Duncan’s multiple comparison test at a *p* < 0.05 level. Numbers in brackets refer to the percentage of B in the entire system.

Soluble B, adsorbed B, and duckweed accumulated B were considered to be the major forms of B in the system. Since soluble B was added to the system in the form of boric acid, soluble B was the main fraction in most treatments. Under severe salt stress, some duckweed roots fell off, broke into pieces, and even decomposed to generate particles with a greater surface area for B adsorption. In addition, the living fronds and roots of *L. minor* also provided surfaces for adsorption. Under severe salt stress (e.g., 150 and 200 mM NaCl), duckweed suffered more damage and consequently dropped more roots and then formed more B-adsorbed particles. It was observed that the turbidity of the water with 150 and 200 mM NaCl increased, which was due to the decomposition of dropped duckweed tissue and the formation of organic colloids. These colloids adsorbed part of the soluble B and then reduced the water-soluble B in the system. Overall, mass balance and B distribution analyses indicate that with the increase in salt stress, more soluble B is converted to forms that are difficult to remove. As we did not investigate B partitioning in insoluble parts, it is difficult to elucidate which process played a major role in B retention.

## Conclusions

The growth and B accumulation of *L. minor* were significantly affected by salt stress. Salt stress inhibited the growth and chlorophyll synthesis of *L. minor*, especially in solutions with NaCl concentrations higher than 100 mM. At lower salinities, *L. minor* accumulated B mainly by absorption, which was inhibited by salt stress. At higher salinities, *L. minor* accumulated B mainly by passive diffusion. Our findings suggest that *L. minor* is suitable for removing B from water with low salinity.
